# Dietary Iron Enhances Colonic Inflammation and IL-6/IL-11-Stat3 Signaling Promoting Colonic Tumor Development in Mice

**DOI:** 10.1371/journal.pone.0078850

**Published:** 2013-11-06

**Authors:** Anita C. G. Chua, Borut R. S. Klopcic, Desiree S. Ho, S. Kristine Fu, Cynthia H. Forrest, Kevin D. Croft, John K. Olynyk, Ian C. Lawrance, Debbie Trinder

**Affiliations:** 1 School of Medicine and Pharmacology, Fremantle Hospital, University of Western Australia, Fremantle, Western Australia, Australia; 2 Western Australian Institute for Medical Research, Perth, Western Australia, Australia; 3 Centre for Inflammatory Bowel Diseases, Fremantle Hospital, Fremantle, Western Australia, Australia; 4 Department of Histopathology, PathWest, Fremantle Hospital, Fremantle, Western Australia, Australia; 5 School of Pathology and Laboratory Medicine, University of Western Australia, Perth, Western Australia, Australia; 6 School of Medicine and Pharmacology, Royal Perth Hospital, University of Western Australia, Perth, Western Australia, Australia; 7 Department of Gastroenterology, Fremantle Hospital, Fremantle, Western Australia, Australia; 8 Institute for Immunology and Infectious Diseases, Murdoch University, Murdoch, Western Australia, Australia; 9 Curtin Health Innovation Research Institute, Curtin University, Perth, Western Australia, Australia; Juntendo University School of Medicine, Japan

## Abstract

Chronic intestinal inflammation and high dietary iron are associated with colorectal cancer development. The role of Stat3 activation in iron-induced colonic inflammation and tumorigenesis was investigated in a mouse model of inflammation-associated colorectal cancer. Mice, fed either an iron-supplemented or control diet, were treated with azoxymethane and dextran sodium sulfate (DSS). Intestinal inflammation and tumor development were assessed by endoscopy and histology, gene expression by real-time PCR, Stat3 phosphorylation by immunoblot, cytokines by ELISA and apoptosis by TUNEL assay. Colonic inflammation was more severe in mice fed an iron-supplemented compared with a control diet one week post-DSS treatment, with enhanced colonic IL-6 and IL-11 release and Stat3 phosphorylation. Both IL-6 and ferritin, the iron storage protein, co-localized with macrophages suggesting iron may act directly on IL-6 producing-macrophages. Iron increased DSS-induced colonic epithelial cell proliferation and apoptosis consistent with enhanced mucosal damage. DSS-treated mice developed anemia that was not alleviated by dietary iron supplementation. Six weeks post-DSS treatment, iron-supplemented mice developed more and larger colonic tumors compared with control mice. Intratumoral IL-6 and IL-11 expression increased in DSS-treated mice and IL-6, and possibly IL-11, were enhanced by dietary iron. Gene expression of iron importers, divalent metal transporter 1 and transferrin receptor 1, increased and iron exporter, ferroportin, decreased in colonic tumors suggesting increased iron uptake. Dietary iron and colonic inflammation synergistically activated colonic IL-6/IL-11-Stat3 signaling promoting tumorigenesis. Oral iron therapy may be detrimental in inflammatory bowel disease since it may exacerbate colonic inflammation and increase colorectal cancer risk.

## Introduction

Inflammatory bowel diseases (IBD) are life-long incurable conditions that are increasing in frequency[1] with high morbidity including an increased risk for colorectal cancer (CRC).[2] These cancers are frequently multiple, flat, histologically high-grade and difficult to recognize by colonoscopy with poor long-term prognosis. Whilst the pathogenesis of IBD associated-CRC is unclear, chronic inflammation is thought to be a significant contributor.[3] Intestinal inflammation can result in abdominal pain, intestinal bleeding and diarrhea, and many IBD patients suffer systemic symptoms of malnutrition and anemia. Anemia is the most common systemic complication of IBD[4] and iron deficiency is evident in many IBD patients.[5], [6]

Chronic intestinal inflammation in IBD results in the upregulation of pro-inflammatory cytokines causing damage to the intestinal mucosa resulting in recurrent intestinal blood loss and anemia.[7] These cytokines also contribute to anemia of inflammation,[7], [8] a condition where inflammation causes dysregulation of iron metabolism and inhibition of erythropoiesis. The inflammatory cytokine interleukin (IL)-6 increases the synthesis of liver hepcidin, a major iron-regulatory hormone central to iron homeostasis, resulting in reduced duodenal iron absorption and retention of iron in macrophages and hepatocytes promoting iron storage thus limiting the availability of iron for erythropoiesis.[9] In addition, tumor necrosis factor (TNF), IL-1β and interferon γ (IFNγ) impair erythropoiesis by inhibiting erythroid progenitor cell proliferation and erythropoietin synthesis.[8]

Oral or parenteral iron supplementation is frequently used to treat anemia in IBD. Although oral iron may be beneficial, it may induce oxidative stress contributing to intestinal inflammation and mucosal damage.[10], [11] Furthermore, excess iron can promote oxidative stress-induced carcinogenesis.[12] Numerous population studies have suggested that high dietary iron intake and body iron stores increase CRC risk.[13], [14] Conversely, iron chelation may reduce intestinal oxidative stress in IBD patients.[15] Interestingly, a reduction of body iron stores has been associated with lower risks for numerous human malignancies.[16] The mechanistic link between iron, colorectal inflammation and carcinogenesis, however, remains to be elucidated.

The aim of this study was to examine the effects of dietary iron on the development of colonic inflammation and tumorigenesis in the azoxymethane/dextran sodium sulfate (DSS) mouse model of inflammation-induced colorectal cancer. We found that dietary iron and acute intestinal inflammation synergistically activated colonic IL-6/IL-11-Stat3 signaling and promoted tumorigenesis. Increased intratumoral IL-6 and possibly IL-11 expression suggested that Stat3 activation may contribute to the development of dietary iron induced-colonic tumors. Anemia was also evident, but was not alleviated by dietary iron supplementation. This study indicates that the practice of using oral iron for the treatment of anemia in IBD should be avoided as it is unlikely to improve anemia, may exacerbate colonic inflammation and increase the risk of CRC.

## Materials and Methods

### Ethics statement

This study was undertaken in accordance with the recommendations of the *Australian code of practice for the care and use of animals for scientific purposes* and was approved by the Animal Ethics Committee of the University of Western Australia (RA/3/100674 and RA/3/100972). Colonoscopy and all surgical procedures were performed under anesthesia.

### Mouse model

Female mice (C57BL6; Animal Resources Centre, Murdoch, WA, Australia) were fed, from 4 weeks of age, either a control (0.02% iron) or an iron-supplemented diet (1% carbonyl iron for 6 weeks followed by 0.1% iron thereafter). At 8 weeks of age, mice were administered a single dose of azoxymethane (AOM; 7.4 mg/kg body weight ip; Sigma-Aldrich Pty Ltd, Castle Hill, NSW, Australia) followed by dextran sodium sulfate (DSS; 2% w/v; MP Biomedicals, Seven Hills, NSW, Australia) in the drinking water for various times using a modified method of Becker et al.[17] In acute studies, mice were treated with DSS for 3 to 7 days post-AOM injection whilst in long-term studies, mice were administered 3 cycles of DSS with each cycle consisting of one week on DSS followed by 2 weeks on plain water. Untreated mice were injected with isotonic saline and given plain drinking water. For brevity, untreated mice fed the iron-supplemented or control iron diet will be referred to as Iron and Control mice, respectively, whilst their AOM/DSS-treated counterparts will be referred to as Iron/DSS and Control/DSS mice. At the end of the experiment, blood was collected by cardiac puncture and tissues were perfused *in situ* with 0.9% sodium chloride before removal. Liver and colon were snapped frozen in liquid nitrogen for gene and protein analyses as well as formalin-fixed for histology or frozen in Tissue-Tek optimal cutting temperature (Sakura Finetek, Alphen aan den Rijn, The Netherlands) embedding medium for immunofluorescence studies.

### Hematological parameters

Whole blood (200 µL) was collected into Minicollect® tubes coated with tripotassium ethylenediamine tetraacetic acid (Greiner Bio-One, Kremsmünster, Austria). Hemoglobin (Hb), hematocrit, mean cell hemoglobin (MCH), mean cell volume (MCV), red blood cell (RBC) count, reticulocyte count and white blood cell (WBC) count were measured using a Cell-Dyn Sapphire analyzer (Abbott Diagnostics, North Ryde, NSW, Australia) at PathWest Laboratories, Fremantle Hospital.

### Iron parameters

Plasma transferrin saturation (TS) and liver non-heme iron concentration were measured as previously described.[18] Colonic iron was detected by histology using enhanced Perls' Prussian blue staining according to the method of Brookes et al.[19]

### Lipid peroxidation

The lipid peroxidation marker, F_2_-isoprostane, was measured in the colon by gas chromatography-mass spectrometry using a deuterium-labeled internal standard, as previously described.[20]

### Immunofluorescence

Immunofluorescence was performed on cryosections of the colon fixed in 4% paraformaldehyde in phosphate-buffered saline as previously described.[21] Rabbit anti-ferritin (1∶500, Dako), biotinylated rat anti-F4/80 (1∶50, AbD Serotec, Raleigh, NC), rat anti-IL-6 (1∶100, BD Biosciences, North Ryde, NSW, Australia) and rabbit anti-pStat3 (1∶50, Santa Cruz Biotechnology, Santa Cruz, CA) were detected using anti-rabbit/rat Alexa Fluor 594 (1∶200, Life Technologies, Mulgrave, VIC, Australia), anti-rat Alexa Fluor 488 (1∶200, Life Technologies) or streptavidin-Alexa Fluor 488 conjugate (1∶800, Life Technologies) and mounted with Long Gold antifade reagent containing 4′,6-diamidino-2-phenylindole (Life Technologies) for nuclear visualization.

Terminal deoxynucleotide transferase dUTP nick end labeling (TUNEL) was performed to detect cellular apoptosis on cryosections of colonic Swiss roll preparations (∼5 cm from anus; rolled from distal to proximal end) that had undergone 1% paraformaldehyde fixation, using the Apoptag® Fluorescein Direct In Situ Apoptosis Detection Kit (Merck Millipore, Kilsyth, VIC, Australia) according to the manufacturer's instructions. TUNEL positive cells and the number of crypts (detected by 4′,6-diamidino-2-phenylindole counterstaining) were determined from merged pictures of sequentially captured images of each fluorophore at 20× magnification from the entire colonic Swiss roll of each mouse. TUNEL positive cells were expressed per 100 crypts for each sample.

### Colonic inflammation and tumor development

Colonic inflammation and tumor development were monitored using a high-resolution mouse video endoscopy. Colonic (proximal and distal) inflammation was assessed using a modified murine endoscopic score of colitis severity (MEICS), examining changes in colonic wall translucency, vascular pattern, presence of fibrin, mucosal granularity and stool consistency.[17] Histological scoring of colonic inflammation (colitis score) using hematoxylin and eosin-stained distal sections of the colon was performed by a histopathologist blinded to the treatment groups and quantified according to a modified method of Dieleman et al.[22] The severity and extent of colonic inflammation as well as crypt damage were assessed. The number and size of colonic tumors were also determined by colonoscopy with tumor size graded from 1 to 5 based on the ratio of tumor coverage of the colonic circumference as described previously by Becker and colleagues.[17], [23] Tumor score was calculated from the sum of size of all tumors in each mouse whilst average tumor size was determined from the division of the tumor score by the number of tumors in each mouse.

### RNA expression

Total RNA was isolated from colonic tissue and reverse-transcribed using Superscript III (Life Technologies) as described previously.[24] Gene transcript levels of cytokines, TNF, IFNγ, IL-6, IL-11 and IL-1β, cell cycle regulators, cyclin D1, cyclin B1 and c-myc, iron transporters, divalent metal transporter 1 (Dmt1), transferrin receptor 1 (Tfr1) and ferroportin (Fpn), and housekeeping genes, hypoxanthine-guanine phosphoribosyltransferase (Hprt) and acidic ribosomal phosphoprotein PO (Arbp), were measured by real-time polymerase chain reaction using a FastSyBR mastermix (Roche Diagnostics, Castle Hill, NSW, Australia). Primer sequences are listed in Table 1. Gene expression was normalized against Hprt or Arbp mRNA expression.

**Table 1:** Mouse primers.

Gene		Primer sequences 5′-3′
TNF	*Forward*	CTGTAGCCCACGTCGTAGC
	*Reverse*	TTGAGATCCATGCCGTTG
IFNγ	*Forward*	ATCTGGAGGAACTGGCAAAA
	*Reverse*	TTCAAGACTTCAAAGAGTCTGAGGTA
IL-6	*Forward*	GTATGAACAACGATGATGCACTTG
	*Reverse*	ATGGTACTCCAGAAGACCAGAGGA
		
IL-11	*Forward*	CTGCACAGATGAGAGACAAATTCC
	*Reverse*	GAAGCTGCAAAGATCCCAATG
IL-1β	*Forward*	GTGGCTGTGGAGAAGCTGTG
	*Reverse*	GAAGGTCCACGGGAAAGACAC
Cyclin D1	*Forward*	CCCTGACACCAATCTCCTCAAC
	*Reverse*	GCATGGATGGCACAATCTCCT
Cyclin B1	*Forward*	ACTTCAGCCTGGGTCGCC
	*Reverse*	ACGTCAACCTCTCCGACTTTAGA
c-myc	*Forward*	TCTCCACTCACCAGCACAACTACG
	*Reverse*	ATCTGCTTCAGGACCCT
Dmt1	*Forward*	TCTATCGCCATCATCCCCACCC
	*Reverse*	TCCACAGTCCAGGAAAGACAGACCC
Tfr1	*Forward*	TTCCTACATCATCTCGCTTAT
	*Reverse*	CATAGTGTTCATCTCGCCGA
Fpn	*Forward*	GTCATCCTCTGCGGAATCATCCTGA
	*Reverse*	GAGACCCATCCATCTCGGAAAGTGC
	*Reverse*	CCAGCAAGCTTGCAACCTTAACCA
Arbp	*Forward*	ACTGGTCTAGGACCCGAGAAG
	*Reverse*	TCCCACCTTGTCTCCAGTCT

### Colonic cytokine release

Colonic explants (5 mm sections taken from distal colon) were cultured in serum free RPMI-1640 containing 25 mM HEPES (Life Technologies) for 24 hours at 37°C. The incubation medium was centrifuged at 20,000 g at 4°C and the explant supernatant collected for cytokine analysis. Colonic IL-6 (R&D Systems Inc., Minneapolis, MN), IL-11 (Sigma-Aldrich) and IL-1β (R&D Systems) concentrations were measured using enzyme-linked immunosorbent assay (ELISA) kits as per manufacturers' instructions.

### Colonic epithelial cell isolation

Whole mouse colons were removed, incised longitudinally and flushed with phosphate buffered saline. The colons were incubated in isolation buffer containing 2 mM ethylenediamine tetraacetic acid (Sigma-Aldrich), 1 mM ethylene glycol tetraacetic acid (Sigma-Aldrich) and 1% fetal bovine serum (Life Technologies) for 30 min at 37°C, with shaking, after which the colons were removed and the incubation solution centrifuged at 300 g for 15 min at 4°C. The supernatant was removed and the resultant cell pellet consisted of the isolated colonic epithelial cells.

### Immunoblotting

Protein from distal colonic tissue of mice or isolated colonic epithelial cells was extracted in a lysis buffer [150 mM NaCl, 25 mM Tris-HCl (pH7.5) and 0.5% Triton X-100] containing cOmplete protease (Roche Diagnostics) and PhosStop phosphatase (Roche Diagnostics) inhibitors. Briefly, protein samples (30 µg) were separated on 4–12% gradient gels (Life Technologies) by sodium dodecyl sulfate-polyacrylamide gel electrophoresis and electroblotted onto nitrocellulose membranes (Pall Life Sciences, Somersby, NSW, Australia). Membranes were incubated with phosphorylated signal transducer and activator of transcription 3 (pStat3, 1∶1000; Cell Signaling Technology Inc, Danvers, MA), total Stat3 (Stat3, 1∶1000; Cell Signaling), proliferating cell nuclear antigen (PCNA, 1∶100; BD Biosciences) and actin (1∶2000; Merck Millipore) at 4°C overnight after which they were incubated with the appropriate secondary antibodies conjugated with horseradish peroxidase (1∶2000; Santa Cruz Biotechnology). Protein expression was detected using enhanced chemiluminescence and quantified by densitometry. Protein expression was normalized against actin expression.

### Statistics

Results are expressed as the mean and SEM, n = 3–8. Differences between groups were analyzed using one-way analysis of variance with Tukey's multiple comparison post-test or unpaired Student's t-test (GraphPad PRISM, La Jolla, CA). Differences between groups were defined as being statistically significant for *P*<0.05.

## Results

### Dietary iron increased colonic iron levels and lipid peroxidation

**Figure 1:**
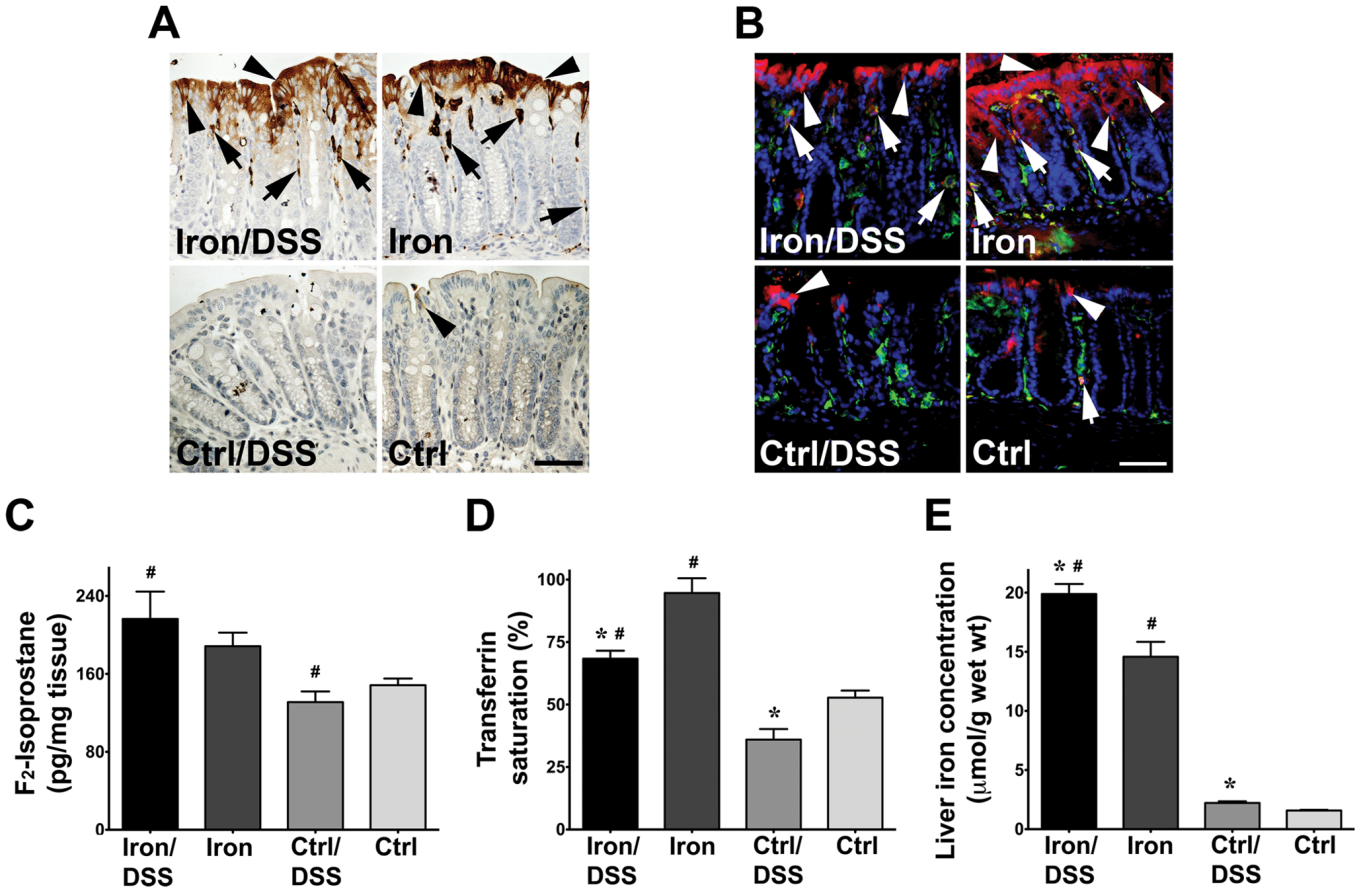
Iron accumulation in the colon increased F_2_-isoprostane levels. 3,3′-diaminobenzidine-enhanced Perls' Prussian blue (A) and ferritin and F4/80 double immunofluorescent (B) staining were performed on colon sections from Iron and Control (Ctrl) mice treated with or without DSS for 3 days. Colonic iron (A) accumulated in apical epithelial cells (black arrowheads) and macrophages of the lamina propria (black arrows). Immunofluorescent staining (B) of ferritin (red) was increased in colonic epithelial cells (white arrowheads) and in lamina propria F4/80^+^ (green) macrophages (white arrows) in iron-supplemented mice. Colon F_2_-isoprostane concentration (C), plasma transferrin saturation (D), liver non-heme iron concentration (E) were measured in Iron and Ctrl mice treated with or without DSS for 7 days. Results are expressed as mean±SEM, n = 4–7. * *P*<0.05 denotes significance between DSS treatment versus no treatment; # *P*<0.05 denotes significance between Iron/DSS versus Ctrl/DSS or Iron versus Ctrl mice. Scale bar denotes 25 µm.

Dietary iron increased iron and ferritin levels in the colon. Iron accumulated in the epithelium and macrophages in the lamina propria of the colonic mucosa in Iron/DSS and Iron mice at day 3 (Fig. 1A). Iron staining was very low or not detected in Control mice (Fig. 1A). Likewise ferritin, an indicator of iron stores, was localized in the colonic epithelium as well as co-localized with F4/80^+^ macrophages in the lamina propria of Iron/DSS and Iron mice (Fig. 1B). Ferritin was also detected in colonic epithelial cells and macrophages in Control/DSS and Control mice but staining was weaker and there were less ferritin-stained cells (Fig. 1B). A similar pattern of co-localization was observed for ferritin and CD11b^+^ or CD68^+^ macrophages (data not shown). Increased iron accumulation in the colon resulted in elevated levels of colonic F_2_-isoprostane, a marker of lipid peroxidation (Fig. 1C; *P*<0.05). DSS treatment alone, however, had no effect on colonic F_2_-isoprostane levels.

Plasma TS was increased by approximately by 2-fold (Fig. 1D; *P*<0.001) and liver iron concentration was increased by approximately 9-fold (Fig. 1E; *P*<0.0001) in mice fed an iron-supplemented diet. At day 7, plasma TS decreased (Fig. 1D; *P*<0.05) and liver iron concentration increased (Fig. 1E; *P*<0.01) in both Iron/DSS and Control/DSS mice compared with Iron and Control mice, respectively.

### Mice developed anemia after DSS treatment

The hematology profile of mice after treatment with DSS for 7 days is presented in Table 2. Hemoglobin, hematocrit, MCH, MCV and RBC count were decreased in Iron/DSS and Control/DSS compared with Iron and Control mice, respectively (*P*<0.05). Conversely, the number of reticulocytes and white blood cells were increased in Iron/DSS and Control/DSS mice (*P*<0.05). There was no difference in these parameters between Iron/DSS and Control/DSS mice. Platelet concentration and MCV were significantly increased in iron-supplemented mice with or without DSS treatment (*P*<0.05). There was a small but significant increase in MCH in Iron mice compared with Control mice (*P*<0.05).

**Table 2:** Hematology profile of Iron and Control mice treated with or without DSS.

Paramaters	Iron/DSS	Iron	Control/DSS	Control
Hb (g/L)	118.9±2.1*	144.3±2.8	122.1±3.5*	137.1±2.8
Hematocrit (%)	37.63±0.50*	45.00±0.82	37.38±0.98*	42.63±0.84
MCH (pg)	15.66±0.14*	16.63±0.09#	15.83±0.05*	16.24±0.12
MCV (fL)	49.50±0.42*#	51.88±0.35#	48.50±0.19*	50.50±0.38
RBC (10^12^/L)	7.61±0.16*	8.65±0.11	7.71±0.23*	8.42±0.16
Reticulocytes (10^9^/L)	639.0±82.4*	354.5±37.9	523.1±30.0*	393.5±17.8
Platelets (10^9^/L)	894.6±30.6#	818.4±33.5#	696.6±12.3	678.9±40.91
WBC (10^9^/L)	5.46±0.64*	3.81±0.21	7.18±0.91*	4.09±0.55

Results are expressed as mean±SEM; n = 7-8. * *P*<0.05; denotes significance between DSS treatment versus no treatment and # *P*<0.05; denotes significance between Iron/DSS versus Control/DSS or Iron versus Control mice. Hb, hemoglobin; MCH, mean cell hemoglobin; MCV, mean cell volume; RBC, red blood cell count; WBC, white blood cell count.

### Iron enhanced DSS-induced colonic inflammation

**Figure 2:**
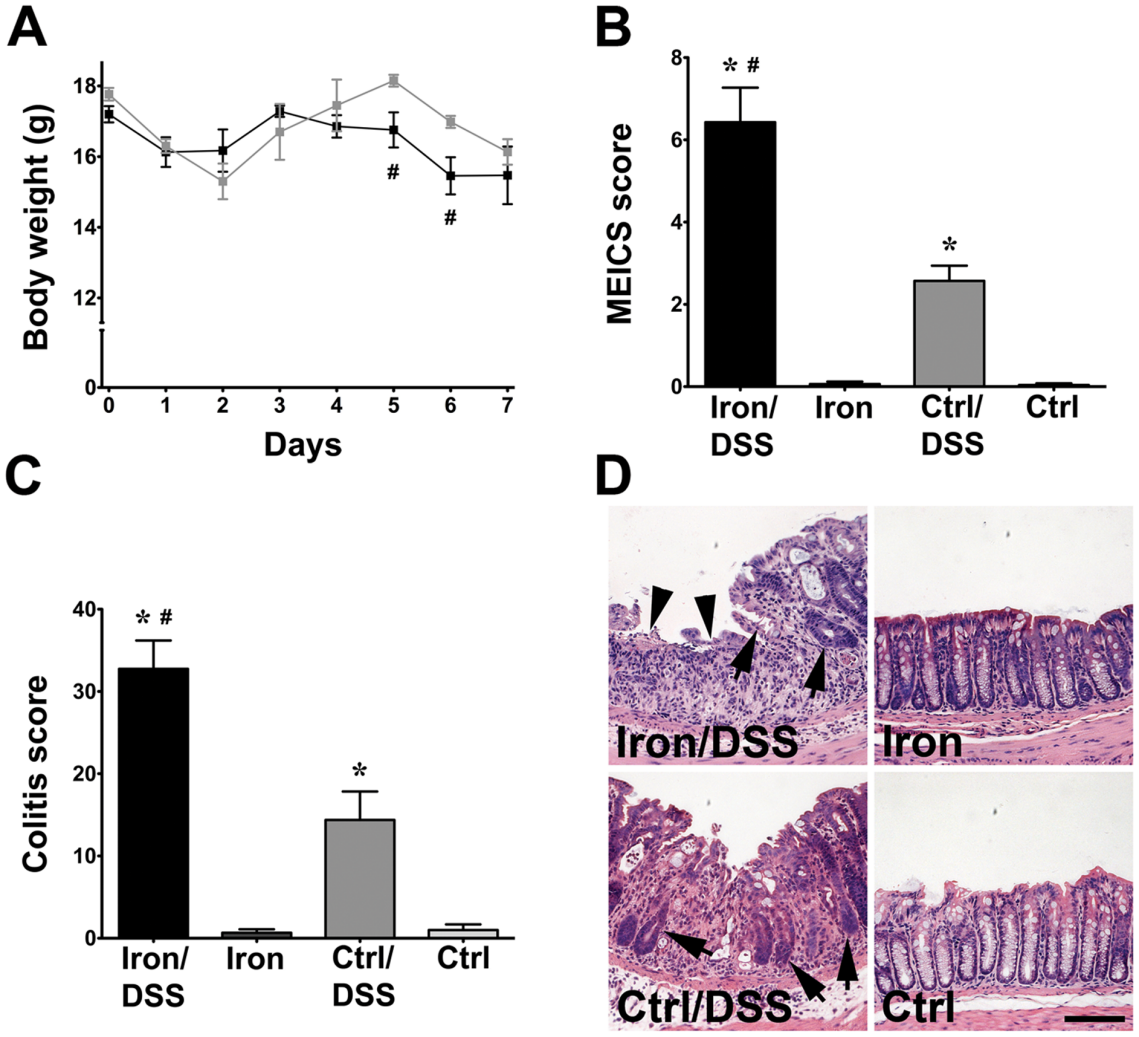
Dietary iron enhanced acute colonic inflammation. Body weight (A), endoscopic MEICS (B) and histological colitis (C) scores were measured in Iron and Control (Ctrl) mice treated with or without DSS for up to 7 days. In hematoxylin and eosin-stained distal colon sections (D), Iron/DSS mice exhibited extensive areas of mucosal erosion (arrowheads) with severe crypt damage (arrows) whilst crypt damage was moderate in Ctrl/DSS mice. There was no damage in Iron and Ctrl mice. Results are expressed as mean±SEM, n = 6–8. * *P*<0.01 denotes significance between DSS treatment versus no treatment; # *P*<0.05 denotes significance between Iron/DSS versus Ctrl/DSS or Iron versus Ctrl mice. Scale bar denotes 50 µm.

Mice treated with DSS exhibited signs of weight loss and diarrhea with or without overt rectal bleeding, reflecting colonic inflammation. The body weight of Iron/DSS mice was lower than Control/DSS mice after days 5 and 6 of DSS treatment (Fig. 2A; *P*<0.05). Colonic inflammation was increased in Iron/DSS and Control/DSS mice compared with Iron and Control mice, respectively. Endoscopically, the MEICS score was significantly higher in Iron/DSS mice than in Control/DSS mice at day 5 (Fig. 2B; *P*<0.05). Similarly, the colitis score was more than 2-fold greater in Iron/DSS mice than in Control/DSS mice at day 7 (Fig. 2C; *P = *0.0001) and is depicted histologically in Fig. 2D. Iron/DSS mice exhibited extensive inflammatory cell infiltration in the colonic mucosa and submucosa with mucosal erosion to greater than 50% of the colonic mucosa with severe loss of crypt structure whilst Control/DSS mice displayed moderate crypt damage with a mainly intact surface epithelium (Fig. 2D). There was no crypt damage observed in Iron and Control mice.

### Iron enhanced colonic IL-6, IL-11 and IL-1β expression and Stat3 signaling

**Figure 3:**
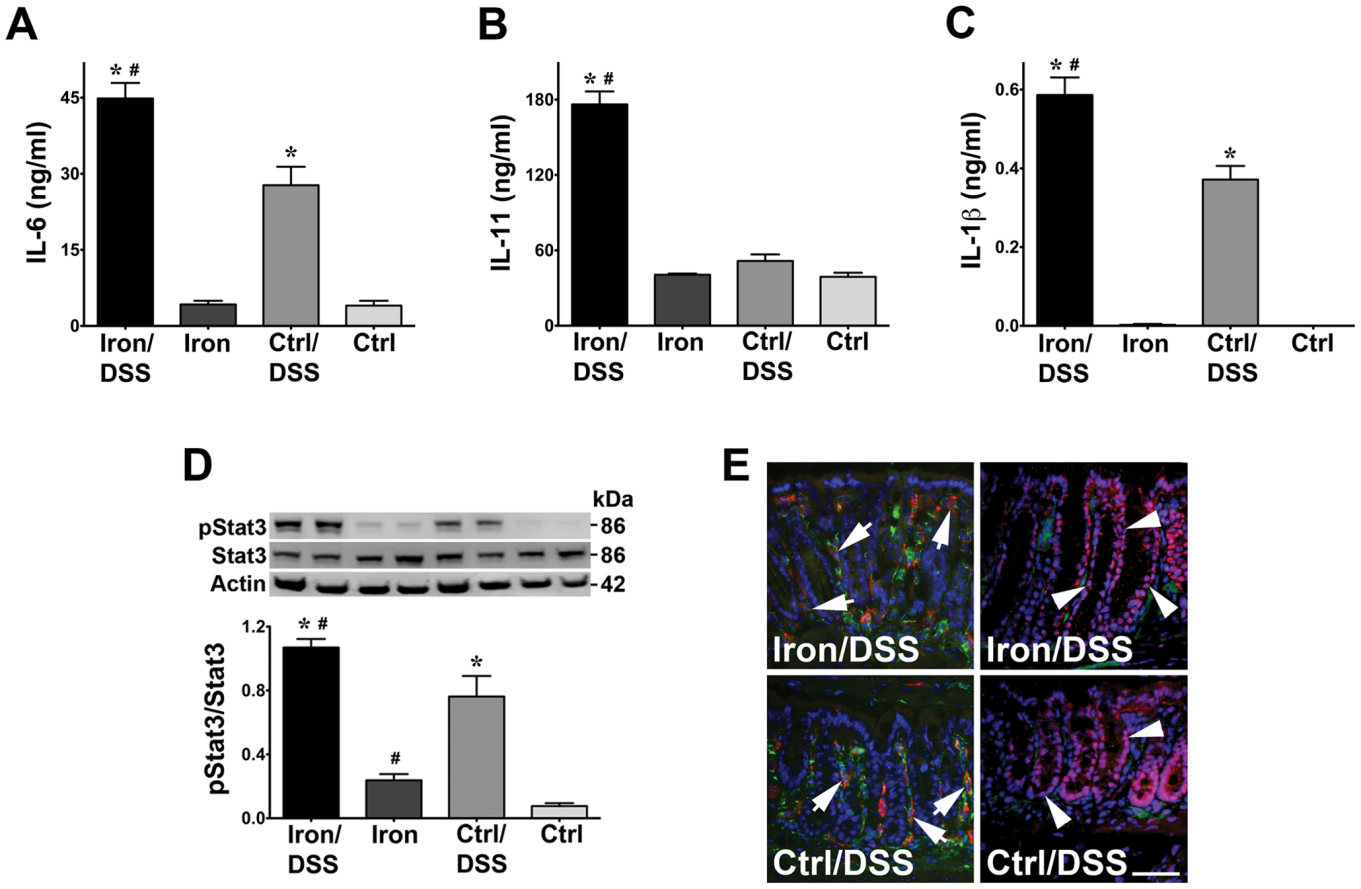
Dietary iron enhanced colonic inflammatory cytokine release and Stat3 signaling during acute colonic inflammation. Colonic IL-6 (A), IL-11 (B) and IL-1β (C) release from colonic tissue explants were measured after 24 hours in culture at 37°C. The tissue explants were harvested Iron and Control (Ctrl) mice treated with or without DSS for 7 days. Colonic Stat3 phosphorylation (D) was also determined in Iron/DSS and Ctrl/DSS mice compared with non-DSS treated mice at day 7. Immunofluorescent staining (E) of F4/80 (green) and IL-6 (red; left panels) or phosphorylated Stat3 (red; right panels) at day 3 of DSS treatment identified F4/80^+^ macrophages (arrows) as a main source of IL-6 production and epithelial crypt cells as active sites of nuclear pStat3 localization (white arrowheads) in Iron and Ctrl mice. Results are expressed as mean±SEM, n = 3–8. * *P*<0.001 denotes significance between DSS treatment versus no treatment; # *P*<0.05 denotes significance between Iron/DSS versus Ctrl/DSS or Iron versus Ctrl mice. Scale bar denotes 25 µm.

Colonic inflammation was associated with increased inflammatory cytokine expression at day 7. Colonic TNF mRNA expression was increased in both Iron/DSS and Control/DSS mice (Table 3; *P*<0.05) compared with non-DSS treated mice whilst IFNγ expression was increased in Iron/DSS mice (*P* = 0.001) with a trend to increase in Control/DSS mice. IL-6 and IL-1β mRNA expression were increased in both Iron/DSS and Control/DSS mice compared with Iron and Control mice, respectively (Table 3; *P*<0.05). There was an increase in mRNA expression of IL-11 (*P*<0.05) but not oncostatin M and ciliary neutrophic factor (data not shown), which are all members of the IL-6 family of cytokines, in Iron/DSS mice compared with Iron mice. Importantly, dietary iron in combination with DSS treatment further increased IL-6, IL-11 and IL-1β mRNA expression (Table 3; *P*<0.05). Similarly, colonic IL-6 and IL-1β released from *ex vivo* colonic tissue were increased in both Iron/DSS and Control/DSS mice (Fig. 3A,C; *P*<0.0001) whilst IL-11 release was increased in Iron/DSS mice (Fig. 3B; *P*<0.0001). Colonic IL-6, IL-11 and IL-1β release were enhanced by iron in DSS-treated mice (Fig. 3A-C; *P*<0.01).

**Table 3:** Cytokine gene expression in Iron and Control mice treated with or without DSS.

Cytokine	Iron/DSS	Iron	Control/DSS	Control
TNF	12.20±3.47 *	1.40±0.36	11.97±3.49 *	1.00±0.22
IFNγ	14.20±2.68 *	0.71±0.17	10.70±6.34	1.00±0.14
IL-6	19.07±4.79 *#	0.60±0.04 #	2.92±0.44 *	1.00±0.16
IL-11	5.35±0.76 *#	1.25±0.10	1.27±0.40	1.00±0.07
IL-1β	17.70±2.04 *#	0.88±0.05	3.16±0.84 *	1.00±0.14

Results are normalized against Hprt or Arbp expression and expressed relative to Control mice as mean±SEM; n = 3–6. * *P*<0.05; denotes significance between DSS treatment versus no treatment and # *P*<0.05; denotes significance between Iron/DSS versus Control/DSS or Iron versus Control mice.

IL-6 and IL-11 activate the transcription factor, Stat3, and colonic Stat3 phosphorylation was increased by both DSS (Fig. 3D; *P*<0.001) and dietary iron (*P*<0.05). Immunofluorescent detection of colonic IL-6 showed that it co-localized with the cell markers, F4/80 (Fig. 3E) and CD11b (data not shown) in DSS-treated mice, suggesting IL-6 was produced by colonic macrophages. Phosphorylated Stat3 protein was predominantly localized in the nucleus of epithelial cells in colonic crypts (Fig. 3E), suggesting the crypt epithelium was the main target of mucosal IL-6/IL-11-Stat3 signaling.

### Iron stimulated cell cycle progression and apoptosis

**Figure 4:**
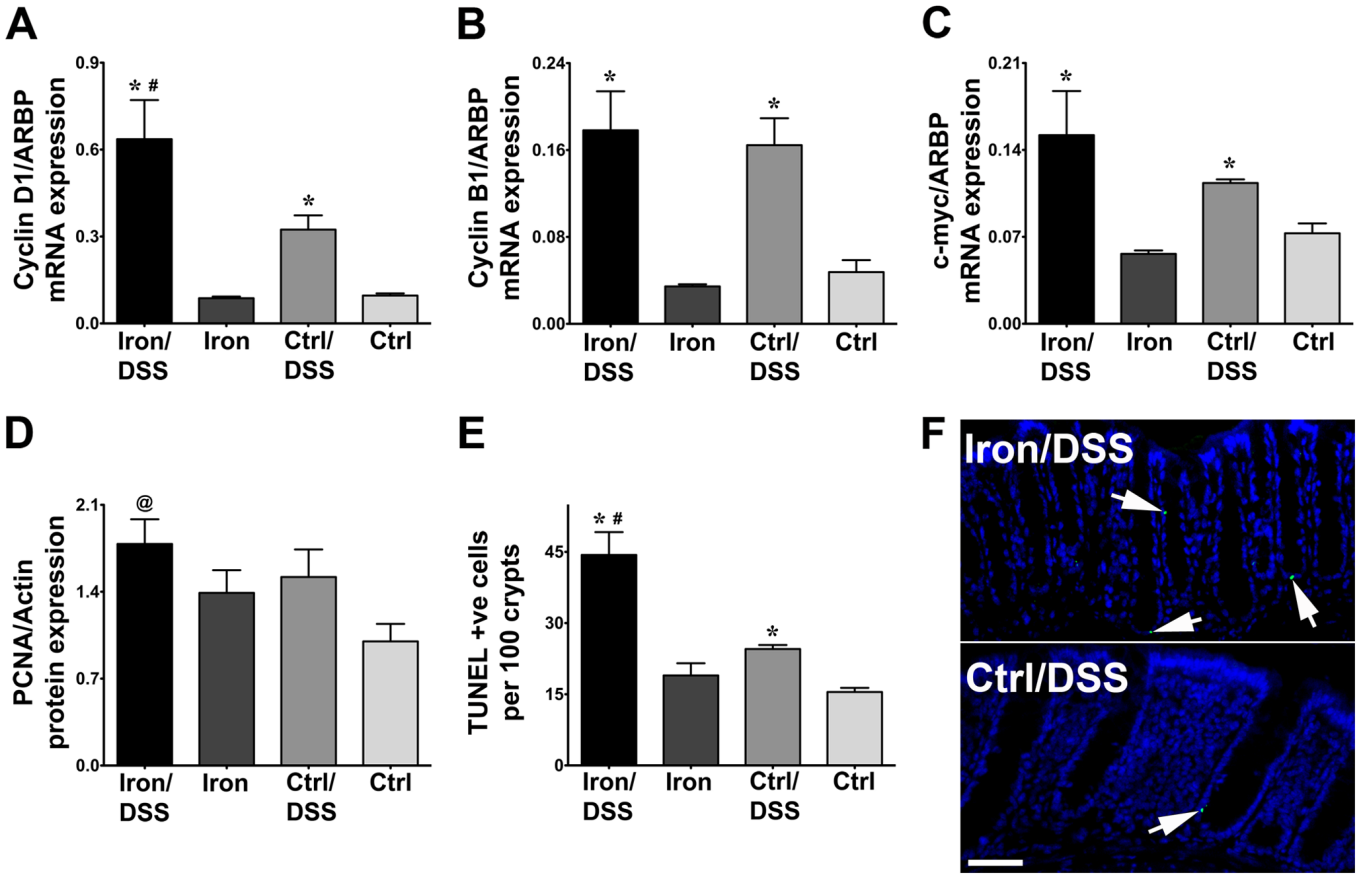
Dietary iron enhanced cyclin D1 expression and promoted epithelial cell apoptosis during acute colonic inflammation. Colonic mRNA expression of cell cycle regulators, cyclin D1 (A), cyclin B1 (B) and c-myc (C) were measured in Iron and Control (Ctrl) mice treated with or without DSS for 7 days. PCNA protein expression (D) was determined by immunoblot in isolated colonic epithelial cells from Iron/DSS and Ctrl/DSS mice compared with non-DSS treated mice at day 3. Apoptotic colonic epithelial cells were quantified (E) from TUNEL staining (F; representative images shown) of whole colonic Swiss roll preparations from Iron and Ctrl mice treated with or without DSS for 3 days. Results are expressed as mean±SEM, n = 3–8. * *P*<0.05 denotes significance between DSS treatment versus no treatment; # *P*<0.05 denotes significance between Iron/DSS versus Ctrl/DSS or Iron versus Ctrl mice. @ *P*<0.05 denotes significance between Iron/DSS versus Ctrl. Scale bar denotes 50 µm.

The expression of genes involved in cell cycle progression was induced in DSS-treated mice. The cell cycle regulators, cyclin D1, cyclin B1 and c-myc mRNA expression were increased in Iron/DSS and Control/DSS mice compared with non-DSS treated mice at day 7 (Fig. 4A-C; *P*<0.05). Cyclin D1 mRNA expression was further increased by dietary iron supplementation (Fig. 4A; *P*<0.05). There was a trend for PCNA protein expression to increase with DSS treatment (Fig. 4D) and expression was significantly increased in Iron/DSS mice compared with Control mice (Fig. 4D; *P*<0.05).

Apoptosis of colonic epithelial cells was evident in DSS-treated Iron and Control mice at day 3 (Fig. 4E,F). Iron/DSS and Control/DSS mice exhibited more TUNEL positive cells compared with their non-DSS treated counterparts (Fig. 4E; *P*<0.01). In addition, there was a 2-fold increase in TUNEL positive cells in Iron/DSS compared with Control/DSS mice (*P*<0.05).

### Iron enhanced colonic tumor development

**Figure 5:**
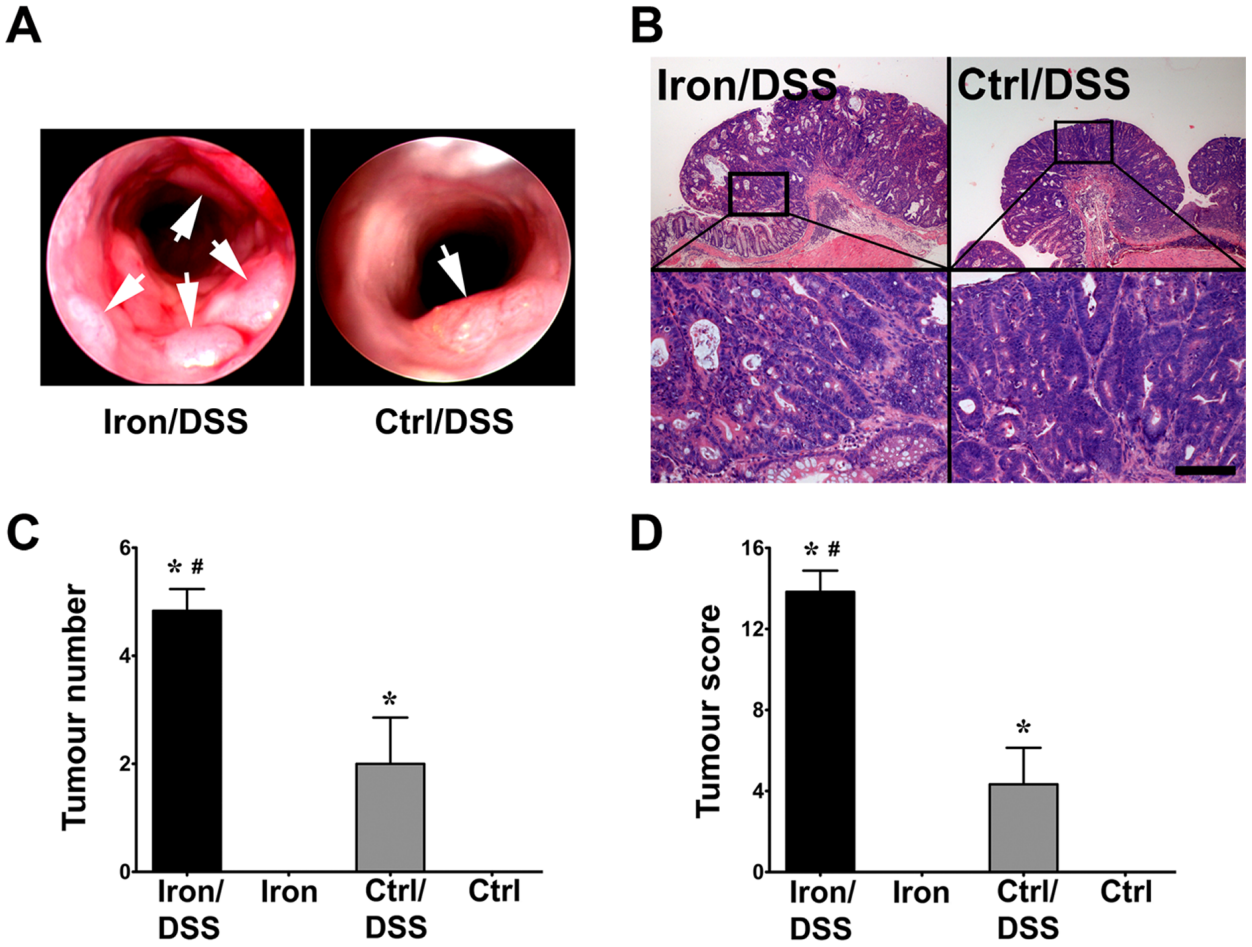
Dietary iron promoted colonic tumor development in inflammation-associated tumorigenesis. Colonoscopy images (A) depict tumor development in Iron/DSS and Control (Ctrl)/DSS mice at day 42. Arrows indicate colonic tumors. Tumors were classified as high-grade dysplasia by hematoxylin and eosin staining (B). Colonic tumor number (C) and tumor score (D) were determined in Iron and Control (Ctrl) mice treated with or without DSS at day 42. Results are expressed as mean±SEM, n = 6. * *P*<0.05 denotes significance between DSS treatment versus no treatment; # *P*<0.05 denotes significance between Iron/DSS versus Ctrl/DSS or Iron versus Ctrl mice. Scale bar denotes 100 µm.

Colonic tumor development was examined by determining the number and size of tumors during colonoscopy (Fig. 5A). Hematoxylin and eosin-stained tumor sections were graded as high-grade dysplasia in Iron/DSS and Control/DSS mice (Fig. 5B). As expected, no tumors were induced in non-DSS treated mice (Fig. 5C,D). By day 42, Iron/DSS mice had developed more colonic tumors (Fig. 5C; *P*<0.05) as well as larger-sized tumors compared with Control/DSS mice (2.88±0.09 versus 2.19±0.11; *P*<0.01) resulting in a higher tumor score (Fig. 5D; *P* = 0.001).

**Figure 6:**
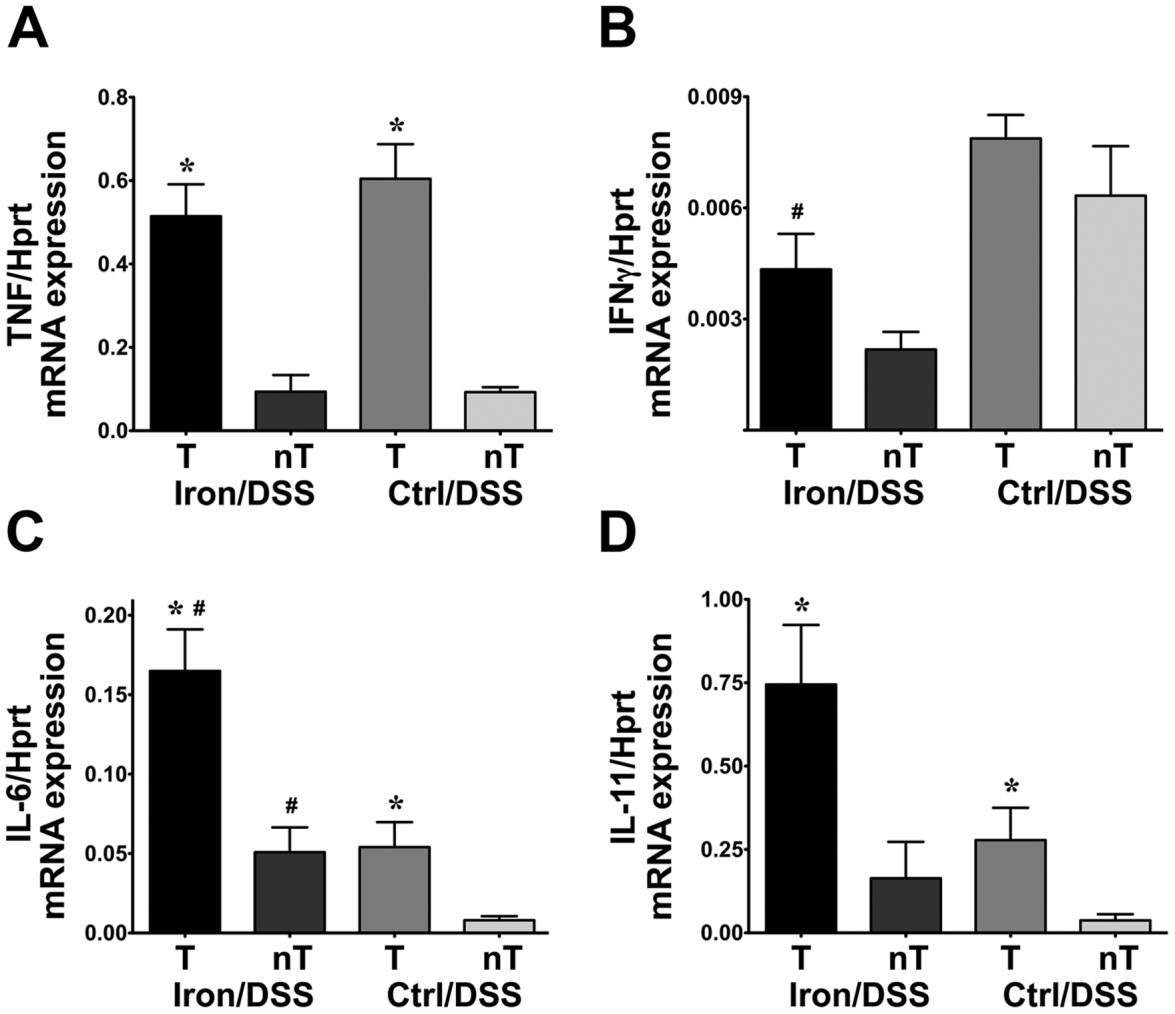
Dietary iron enhanced intratumoral IL-6 gene expression in inflammation-associated tumorigenesis. TNF (A), IFNγ (B), IL-6 (C) and IL-11 (D) mRNA expression were measured in colonic tumor (T) and surrounding non-tumor (nT) tissue from Iron/DSS and Control (Ctrl)/DSS mice at day 78. Results are expressed as mean±SEM, n = 3–6. * *P*<0.05 denotes significance between T and nT tissue from Iron/DSS or Ctrl/DSS mice; # *P*<0.05 denotes significance between Iron/DSS versus Ctrl/DSS in T or nT tissue.

TNF, IFNγ, IL-6 and IL-11 gene expression were higher in non-tumor tissue from Iron/DSS and Control/DSS mice compared with colonic tissue from Iron and Control mice at day 78 (data not shown). Cytokine gene expression was also examined in colonic tumor versus non-tumor tissue; the latter was taken from areas surrounding tumors. TNF mRNA expression was significantly increased in tumor compared with non-tumor tissue in Iron/DSS and Control/DSS mice (Fig. 6A; *P*<0.01) whilst there was no difference between tumor and non-tumor IFNγ mRNA expression (Fig. 6B). There was a trend for IFNγ expression to be downregulated by dietary iron. IL-6 and IL-11 mRNA expression were increased in colonic tumors compared with non-tumor tissue in both Iron/DSS and Control/DSS mice (Fig. 6C,D; *P*<0.05). As in the early phase of DSS induction (Table 3), colonic tumor IL-6 (*P*<0.05) and possibly IL-11 (*P* = 0.07) mRNA expression was higher in Iron/DSS compared with Control/DSS mice but not TNF or IFNγ (Fig. 6A–D).

### Iron transporter gene expression was altered in colonic tumors

**Figure 7:**
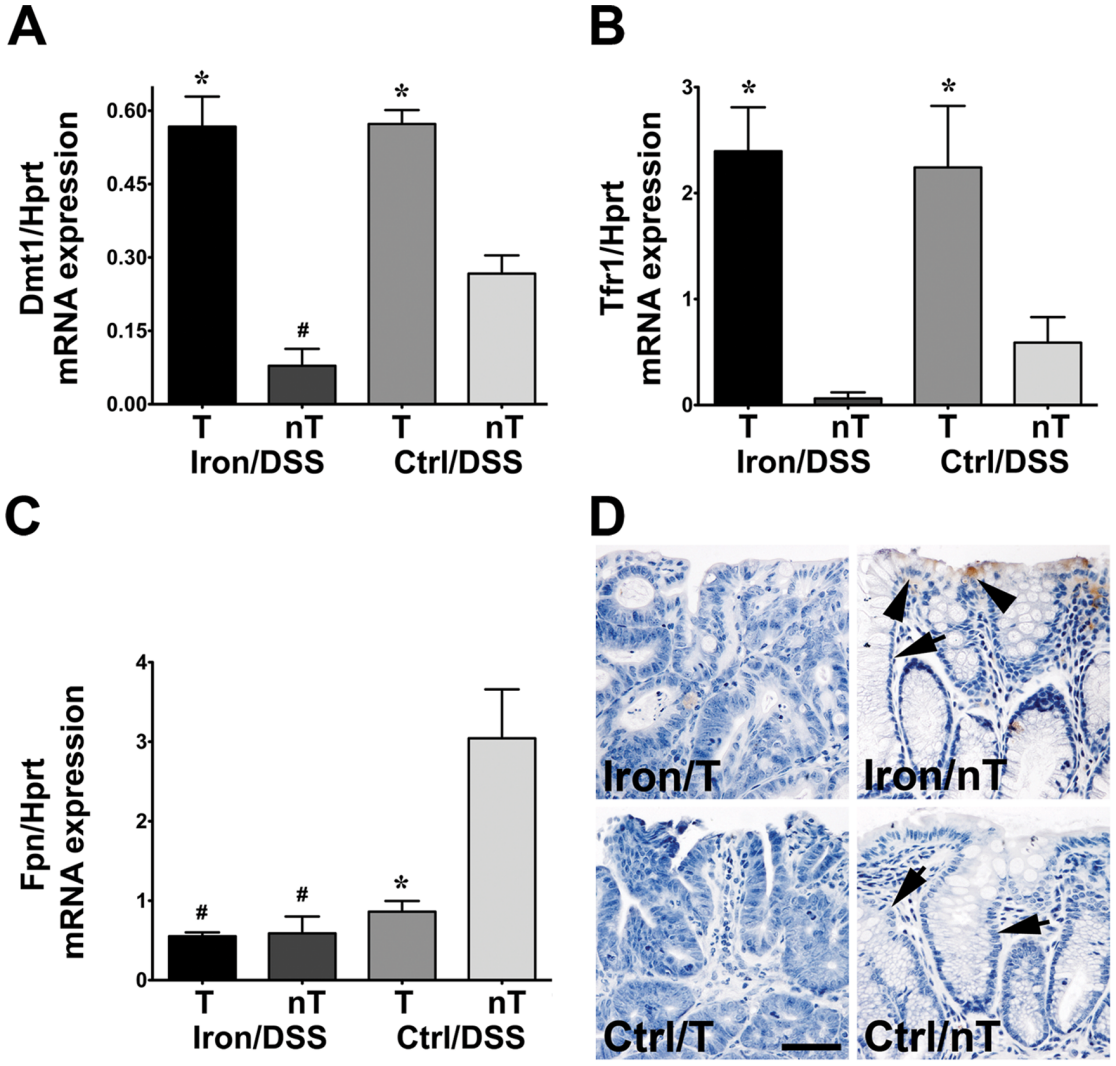
Iron transporter gene expression was modified in colonic tumors in inflammation-associated tumorigenesis. Iron importer, Dmt1 (A) and Tfr1 (B), as well as iron exporter, Fpn (C), mRNA expression were measured in colonic tumor (T) and non-tumor (nT) tissue from Iron/DSS and Control (Ctrl)/DSS mice at day 78. 3,3′-diaminobenzidine-enhanced Perls' Prussian blue staining (D) showed that no iron deposits were detected in the tumor epithelium from Iron/DSS or Ctrl/DSS mice. In surrounding non-tumor tissue, there was some stainable iron detected in colonic epithelial cells (arrowheads) of Iron/DSS mice compared with Ctrl/DSS mice and there was some evidence of distension and branching of glands in the mucosa (D; arrows). Results are expressed as mean±SEM, n = 3–6. * *P*<0.05 denotes significance between T and nT tissue from Iron/DSS or Ctrl/DSS mice; # *P*<0.05 denotes significance between Iron/DSS and Ctrl/DSS in T or nT tissue. Scale bar denotes 25 µm.

Iron transporter gene expression was assessed in colonic tumor and non-tumor tissue. The expression of the cellular iron import genes, Dmt1 and Tfr1, was increased in tumors compared with non-tumor tissue (Fig. 7A,B; *P*<0.05) whilst the expression of the cellular iron export gene, Fpn, was decreased (*P*<0.01) or unchanged (Fig. 7C). In non-tumor tissue, Dmt1 and Fpn mRNA expression were decreased in Iron/DSS compared with Control/DSS mice (Fig. 7A,C; *P*<0.05) whilst there was a trend for Tfr1 expression to decrease (Fig. 7B; *P* = 0.06). In contrast, iron importer expression in colonic tumors was similar in Iron/DSS and Control/DSS mice (Fig. 7A,B). Tumor Fpn mRNA expression was lower in Iron/DSS compared with Control/DSS mice (Fig. 7C; *P*<0.05).

In long-term studies, iron staining in the colon of Iron mice was similar to that observed in the acute studies (Fig. 1) with iron accumulating in the epithelium and macrophages in the lamina propria of the colonic mucosa (data not shown). In Iron/DSS mice, there was weak but stainable iron in the mucosa of non-tumor tissue that was absent in Control/DSS mice (Fig. 7D). In contrast, there was no stainable iron detected in colonic tumor epithelium and stroma in both Iron/DSS and Control/DSS mice (Fig. 7D). In non-tumor tissue, there was evidence of architectural crypt distortion with distension and branching of glands (Fig. 7D).

## Discussion

In the present study, dietary iron enhanced colonic IL-6/IL-11-Stat3 signaling and promoted colonic inflammation and tumor development in a DSS mouse model of inflammation-associated colorectal tumorigenesis. There were synergistic effects of iron and colonic inflammation on IL-6 and IL-11 expression as well as Stat3 phosphorylation indicating iron activated Stat3 signaling. Both ferritin and IL-6 co-localized with macrophages in the lamina propria suggesting that these cells accumulate iron as well as synthesize IL-6. Intratumoral IL-6 and possibly IL - 11 expression was further increased by dietary iron suggesting that iron-induced colonic tumorigenesis may be mediated by IL-6/IL-11-Stat3 signaling. We also report the presence of anemia of inflammation and iron deficiency anemia in this mouse model that was not alleviated by dietary iron supplementation.

Liver iron concentration and plasma TS were increased in iron-supplemented mice confirming iron loading conferred by dietary iron supplementation (Fig. 1E,F). Iron accumulated in the colonic epithelium as well as in mucosal macrophages in iron-supplemented mice. Increased levels of colonic F_2_-isoprostane, a marker of lipid peroxidation, in iron-supplemented mice indicated the presence of oxidative stress as previously reported,[25] whilst colonic inflammation alone had no effect on lipid peroxidation (Fig. 1C). Decreased TS, Hb, hematocrit, MCH, MCV and RBC as well as increased reticulocyte count in both Iron/DSS and Control/DSS mice (Fig. 1D; Table 2) were consistent with the presence of microcytic, hypochromic iron deficiency anemia and increased erythropoietic activity in the bone marrow of DSS-treated mice. Interestingly, the platelet concentration was increased in mice fed the iron-supplemented diet suggesting that iron may influence thrombopoietin, the hormone that regulates platelet production, whilst the WBC count was elevated in DSS-treated mice reflecting the colonic inflammation. Colonic inflammation was also associated with an increase in liver iron concentration and a reduction in plasma TS (Fig. 1D,E). The observed anemia following DSS treatment was most likely due to intestinal blood loss that was exacerbated by the anemia of inflammation which is associated with hypoferremia as a result of liver iron retention limiting the availability of iron for erythropoiesis.[8] The presence of iron deficiency anemia and anemia of inflammation in DSS-treated mice mirrors the anemia observed in IBD patients[7] and did not improve with dietary iron supplementation.

The DSS-induced colonic inflammation in the mice was associated with extensive infiltration by inflammatory cells (Fig. 2D) consisting predominantly of macrophages and neutrophils.[26] As expected, colonic expression of the pro-inflammatory cytokines, TNF, IFNγ, IL-6 and IL-1β (Table 3; Fig. 3A,C), was increased consistent with other colitis models[27], [28] and IBD colitis.[26], [29], [30] The colonic inflammation was exacerbated by dietary iron as observed in other rodent DSS studies.[10], [31] Colonic IL-6, IL-11 and IL-1β levels were also synergistically increased with DSS treatment and dietary iron supplementation (Table 3; Fig. 3A-C). As IL-6 and IL-11 are activators of the transcription factor, Stat3, intracelluar signaling is initiated when they bind to their respective receptors and associate with gp130 triggering Stat3 activation via Jak[32] within the colonic mucosa.[30], [33], [34] Consistent with this, colonic Stat3 was activated in the DSS-treated mice with dietary iron supplementation augmenting colonic Stat3 activation. IL-6 and IL-11 as well as IL-1β are also downstream target genes of Stat3 and may contribute to the inflammation in the mice.[35] Our findings thus demonstrate that dietary iron not only exacerbates colonic inflammation but also promotes IL-6/IL-11-Stat3 signaling.

Stat3 also regulates genes involved in cell growth, proliferation, apoptosis, survival and migration[35] and studies in genetically modified mice with impaired Stat3 signaling highlight the integral role of Stat3 in intestinal epithelial cell regeneration.[36], [37] In our study, an upregulation of cell proliferation gene expression in the presence of colonic inflammation was observed (Fig. 4A-C). Dietary iron further increased cyclin D1 expression suggesting that iron may drive the G1 to S transition phase of the cell cycle during inflammation. This was supported by iron chelation studies where cellular iron depletion resulted in the downregulation of cyclin D1 expression.[38], [39] Colonic epithelial cell apoptosis was also increased with acute DSS treatment and this was enhanced by iron (Fig. 4E,F). Higher apoptotic activity in the epithelium of Iron/DSS mice was consistent with the more severe colonic tissue damage and inflammation observed in these mice (Fig. 2). Elevated colonic lipid peroxidation in the Iron/DSS mice (Fig. 1C) suggests that enhanced apoptosis in these mice may be due to increased oxidative stress and this may exacerbate colonic inflammation by further compromising mucosal epithelial barrier integrity thereby allowing transmigration of gut microflora into the mucosa. Colonic inflammation has been shown to increase epithelial cell proliferation and apoptosis, as well as induce oxidative stress in animal colitis models and IBD colitis,[40]–[45] whilst increasing anti-oxidant activity can attenuate the inflammation.[46], [47] Furthermore, dietary iron supplementation promotes and iron deprivation ameliorates colitis-induced oxidative stress.[10], [15], [25]

Chronic colonic inflammation increases the risk of CRC in ulcerative colitis and Crohn's disease, correlating with disease duration, extent and severity of inflammation.[2] Unresolved intestinal inflammation can lead to continuous excessive inflammatory cytokine production and persistent STAT3 and/or NFκB activation which may promote carcinogenesis by switching on tumor cell survival pathways.[35], [48] Inhibition of IL-6, IL-11 or Stat3 signaling can suppress the development of inflammation-associated CRC, with IL-11-Stat3 signaling playing a more prominent role than IL-6 in colonic tumorigenesis.[36], [37], [49] In the present study, IL-6, IL-11 and TNF mRNA expression were increased in colonic tumors in Iron/DSS and Control/DSS mice (Fig. 6A,C,D) consistent with other reports.[50]–[52] Dietary iron increased the number and size of DSS-induced colonic tumors and enhanced intratumoral IL-6, and possibly IL-11, expression (Fig. 6C,D) suggesting that iron promotes colonic tumorigenesis via the Stat3 signaling pathway. The mechanism underlying iron promotion of colonic IL-6/IL-11-Stat3 signaling is not known. Recently, it has been suggested that iron may modulate Toll-like receptor 4 (TLR4) signaling and IL-6 production.[53] TLR4 expression is upregulated in IBD[54] and promotes inflammation-associated colorectal tumorigenesis in mice.[55] As both IL-6 and IL-11 activate Stat3, and in turn are regulated by Stat3 and/or NFκB,[35], [56] it is likely that iron and colonic inflammation may regulate IL-11 in a similar manner to IL-6.

Iron is important for DNA synthesis and cell cycle progression, hence, tumor cells have a high requirement for iron to sustain their proliferation.[57] The expression of iron importers, Dmt1 and Tfr1, increased and iron exporter, Fpn, decreased in colonic tumors of DSS-treated mice (Fig. 7A-C) suggesting that there was increased iron uptake by the tumors. Dmt1, Tfr1 and Fpn protein expression have been shown to be upregulated in mouse and human colorectal adenoma and/or carcinoma.[19], [58] Downregulation of Fpn expression, however, has been reported in other cancers and is associated with a poor prognosis in breast cancer.[59] Iron staining in the colonic tumors was not detectable (Fig. 7D) suggesting that the increased iron taken up by the tumors did not accumulate but was instead utilized for cell proliferation.

Dietary iron has been shown to promote colonic inflammation and tumor development in other DSS models whilst parenteral iron administration has no effect.[10], [60] This suggests that increased iron levels in the intestinal lumen are required for exacerbation of colonic inflammation. Only 5–10% of daily dietary iron intake is absorbed, mainly by the small intestine and the remaining iron will reach the colon where some of the iron is absorbed. The route of iron administration to treat the anemia in IBD patients is, therefore, important since dietary iron could exacerbate intestinal inflammation and potentially increase the risk of tumor development. Indeed, in a mouse model of ileitis, mice fed a low iron diet exhibited less severe inflammation compared with mice fed an iron replete diet.[61]

In conclusion, dietary iron and colonic inflammation synergistically activated colonic IL-6/IL-11-Stat3 signaling promoting tumorigenesis. Increased intratumoral IL-6 and possibly IL-11 expression suggested that dietary iron may promote colonic tumor development via a Stat3-mediated pathway. This study also suggests that the use of oral iron for the treatment of anemia in IBD should be avoided as it is unlikely to improve anemia and may exacerbate colonic inflammation increasing the risk of CRC.
